# Supplementary material for: Using Relational Community Engagement within the Digital Health Intervention (DHI) to Improve Access and Retention among People Living with HIV (PLWH): Findings from a Mixed-Method Study in Cambodia
Source: Int J Environ Res Public Health. 2023 Mar 23;20(7):5247. doi: 10.3390/ijerph20075247 (PMC10093806; doi:10.3390/ijerph20075247)
Supplement: Supplementary file 1 [file ijerph-20-05247-s001.zip › ijerph-2285214-supplementary.pdf]

Section 2. Assessment on DHI usage and complementary relational skill training

Table S1

ITEM	Circle number you most agree with<br>1- Strongly disagree<br>2- Disagree<br>3- Neither agree nor disagree<br>4- Agree<br>5- Strongly agree<br>Or replay as applicable				
**2.1. Uses of DHI**					
1. The DHI provided by the project greatly improved our ability to keep in touch with certain patients, even after the COVID-19 situation had improved	**1**	**2**	**3**	**4**	**5**
2. Many of our patients could benefit from DHI that the project is offering to care providers	**1**	**2**	**3**	**4**	**5**
3. Many of our patients have the ability to be connected through DHI provided by the project	**1**	**2**	**3**	**4**	**5**
4. Many of our patients who have the ability to be connected through DHI provided by the project are using these technologies to connect with their care providers	**1**	**2**	**3**	**4**	**5**
5. Using DHI to keep in touch with patients should remain as one of the options even after the COVID-19 situation had improved	**1**	**2**	**3**	**4**	**5**
**2.2. Usefulness of relational CE training**					
6. Relational CE training sessions within the DHI had been very useful for me	**1**	**2**	**3**	**4**	**5**
7. Four sessions (of relational skill training) during the intervention period of about two months seemed to be adequate (for me)	**1**	**2**	**3**	**4**	**5**
8. I believe the relational CE training sessions helps me improve my communication and build more trust with my patients	**1**	**2**	**3**	**4**	**5**
9. I have developed some relational skills I considered useful in order to effectively and compassionately communicate with patients from these training sessions	**1**	**2**	**3**	**4**	**5**
10. I understood most of the contents that had been taught or discussed during the relational CE training sessions	**1**	**2**	**3**	**4**	**5**

11. I am confident in using the skills I have learned from these sessions when engaging with patients in the future	**1**	**2**	**3**	**4**	**5**
12. If you have to pick only one session out the four sessions that you considered the most useful in your line of work, which session would that be?	Session number: …….				
13. If you are given another chance to undergo any session one more time, which session would you want to undergo again?	Session number: …….				
**2.3. Perceived need of relational skills and their other applications**					
14. I find a lot of uses for these training sessions while engaging with patients using DHI	**1**	**2**	**3**	**4**	**5**
15. I find a lot of uses for these training sessions in the future with my patients even out of the context of DHI	**1**	**2**	**3**	**4**	**5**
16. I see myself using a lot of the things I learned during these training sessions in the future	**1**	**2**	**3**	**4**	**5**
17. I am very likely to recommend other providers to undergo these relational training sessions in order to improve their relational skills	**1**	**2**	**3**	**4**	**5**
18. Name the <u>one skill</u> that you have learned during the training sessions that you find to be most useful	………………………………………………………………………				
19. Name the <u>one skill</u> that you wish you could have more time learning	………………………………………………………………………				
20. Other comments or suggestions (if any)	………………………………………………………………………<br>………………………………………………………………………<br>………………………………………………………………………<br>………………………………………………………………………<br>………………………………………………………………………				
**Thank you for participating in our study**					
